# Bridging the clinical gaps: genetic, epigenetic and transcriptomic biomarkers for the early detection of lung cancer in the post-National Lung Screening Trial era

**DOI:** 10.1186/1741-7015-11-168

**Published:** 2013-07-19

**Authors:** John F Brothers, Kahkeshan Hijazi, Celine Mascaux, Randa A El-Zein, Margaret R Spitz, Avrum Spira

**Affiliations:** 1 Bioinformatics Program, Boston University, Boston, MA, USA; 2 Section of Computational Biomedicine, Department of Medicine, Boston University School of Medicine, Boston, MA, USA; 3 Department of Medical Oncology, Princess Margaret Cancer Centre, University of Health Network, University of Toronto, Toronto, Ontario, Canada; 4 Dan L Duncan Cancer Center, Baylor College of Medicine, Houston, TX, USA

**Keywords:** Biomarker, Diagnostics, Early detection, Epigenetics, Genetics, Lung cancer, Screening, Transcriptomics

## Abstract

Lung cancer is the leading cause of cancer death worldwide in part due to our inability to identify which smokers are at highest risk and the lack of effective tools to detect the disease at its earliest and potentially curable stage. Recent results from the National Lung Screening Trial have shown that annual screening of high-risk smokers with low-dose helical computed tomography of the chest can reduce lung cancer mortality. However, molecular biomarkers are needed to identify which current and former smokers would benefit most from annual computed tomography scan screening in order to reduce the costs and morbidity associated with this procedure. Additionally, there is an urgent clinical need to develop biomarkers that can distinguish benign from malignant lesions found on computed tomography of the chest given its very high false positive rate. This review highlights recent genetic, transcriptomic and epigenomic biomarkers that are emerging as tools for the early detection of lung cancer both in the diagnostic and screening setting.

## Review

### Introduction

Lung cancer is the leading cause of cancer death in both men and women in the US and the world, causing more than 1 million deaths per year [1-4]. The global cancer burden in annual cases is projected to double by 2050, and lung cancer is expected to remain the leading cause of all cancer deaths during that time. Cigarette smoke remains the main risk factor for lung cancer, with 85% to 90% percent of lung cancer cases in the US occurring in current or former smokers. However, only 10% to 20% of heavy smokers develop lung cancer [5]. While smoking cessation gradually reduces the risk of lung cancer, the majority of new lung cancer cases occur in former smokers. The high mortality in patients with lung cancer (80% to 85% in five years) results in part from our inability to predict which of the 100 million current and former smokers in the US are at greatest risk for developing lung cancer, and from the lack of effective tools to diagnose the disease at an early stage [6].

**Figure 1:**
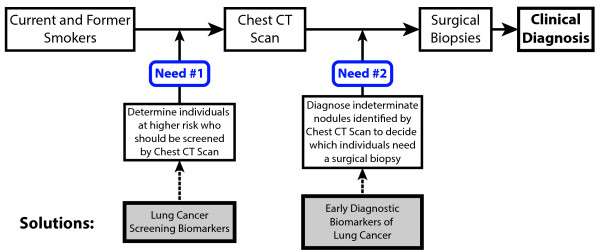
**An overview of clinically unmet needs that exist following the National Lung Screening Trial.** While there is a reduction in both lung cancer mortality and all-cause mortality when using low-dose CT, there are still two major unmet needs highlighted by the trial. The first is the need to limit the number of people who are screened with low-dose CT to those with the highest risks. Genetic, transcriptomic and epigenetic screening biomarkers could meet this need by identifying smokers with the highest likelihood of developing lung cancer. The second unmet need comes from the high number of nodules identified by CT, which are false positives for lung cancer. Early diagnostic biomarkers could play a key role in identifying which nodules are likely to be cancerous before sending patients into surgery.

Recent results published from the National Lung Screening Trial have shown that screening high-risk smokers (based on age and cumulative exposure to tobacco smoke) with low-dose helical computed tomography (CT) can lead to a reduction in both lung cancer mortality (by 20.0%) and all-cause mortality (by 6.7%) compared to standard radiographic screening. While this landmark study is already impacting CT screening guidelines and practices across the US, there were a number of important caveats to the study, including the age (55 to 75 years old) and smoking exposure cutoffs (>30 pack-years) chosen for patient inclusion, and the duration of annual CT screening (three years). Importantly, 39.1% of all participants in the low-dose CT arm of the trial had at least one positive screen for lung cancer, and 96.4% of these initial positive screenings represented false positives for lung cancer [7]. This overabundance of false positives could lead to higher screening costs and unnecessary invasive procedures on many smokers who do not actually have lung cancer. Thus, there is a critical need to develop biomarkers that can determine which of the frequently detected lung nodules on CT scan are malignant (that is, diagnostic markers), and to further define the large high-risk population that would be eligible for screening by CT to increase the efficacy of screening and to reduce the cost and morbidity associated with it (that is, screening markers; Figure 1).

**Figure 2:**
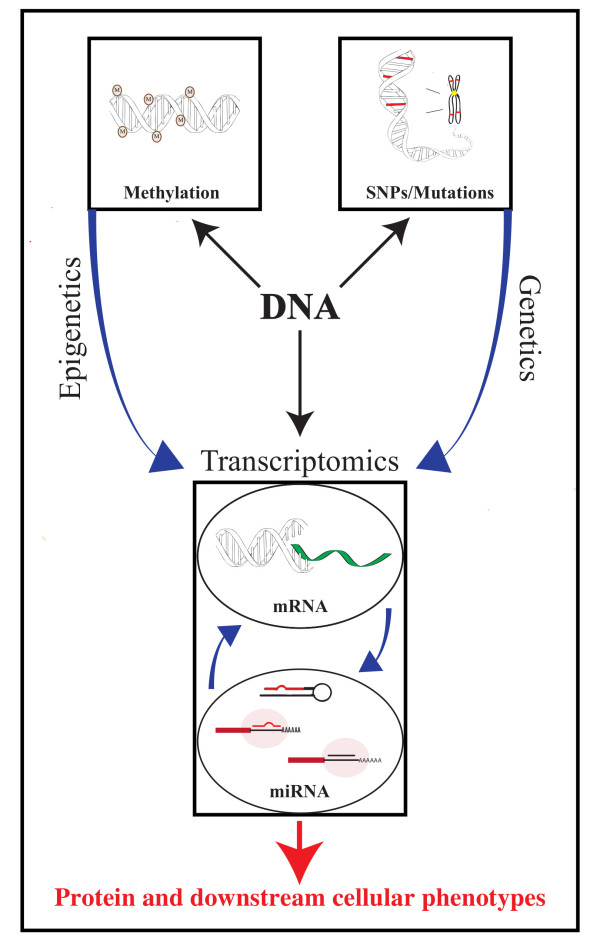
**Biological rationale for addressing clinical issues by using upstream early events that ultimately lead to lung cancer phenotypes as genomic biomarkers.** The diagram highlights early upstream markers for diagnosing or screening of lung cancer far in advance of the development of clinically evident invasive carcinomas, which are mainly driven by genetic, epigenetic and transcriptomic damage.

The sequencing of the human genome together with the technological advances that enabled this accomplishment have ushered in a new era of molecular biomarker development that promises to help address these unmet needs. This review will summarize recent genetic, transcriptomic and epigenomic biomarkers that are emerging as tools for the early detection of lung cancer (Figure 2), both in the diagnostic and the screening setting (prognostic and predictive biomarkers will not be covered). The review will focus on genome-wide studies in clinical biospecimens (no animal models or cell line studies) that leverage these emerging high-throughput technologies, and will review commonality of variants between lung cancer and chronic obstructive airways disease. Although there are a number of promising metabolic and proteomic biomarkers for early lung cancer detection, these fall outside the scope of this review [8].

### Genome-wide association studies to identify genetic risk factors for lung cancer

Initial genome-wide associations in lung cancer robustly implicated SNPs (Table 1) spanning the chromosome 15q25 region encoding the gene cluster of nicotinic receptors, *CHRNA3/A5/B4*[9-12]. Subsequent multi-investigator consortia analyses confirmed the association of SNPs spanning this region with heavy smoking, nicotine dependence, craving and related endophenotypes [11,13,14]. Saccone *et al*. [13] conducted a meta-analysis across 34 datasets of European-ancestry participants (Table 1), including a diverse group of 38,617 smokers, and demonstrated that rs16969968, a nonsynonymous coding polymorphism of the *CHRNA5* gene, correlated highly significantly with smoking behavior (odds ratio = 1.33, *P* = 5.96 × 10^−31^). Three other large smoking genetics consortia confirmed this locus as that most associated with smoking quantity [11,14,15].

**Table 1:** Regions and genes associated with lung cancer and/or chronic obstructive pulmonary disease

**Reference**	**Gene**	**Chromosome region**	**Population size**	**Platform**
[9]	*CHRNA5*, *CHRNA3* and *CHRNB4*, *HTERT*,	15q24-25.1	Discovery 1,154 Cases 1,137	Illumina Hapmap 300
	*CLPTM1L*	5p15.33	Replication two sets- Texas 711/632 and UK 2,013/3,062 Caucasian	
[10]	*CHRNA5*, *CHRNA3* and *CHRNB4*	15q24-25.1	Discovery 1,989/2,513/4,752 Caucasian	Illumina Hapmap 300
	*HTERT*, *CLPTM1L*	5p15.33		
	HLA region	6p21		
[11]	*CHRNA5*, *CHRNA3* and *CHRNB4*	15q24-25.1	1,024/32,244 Caucasian	Illumina (Human Hap300 and Human Hap300-duo + Bead Arrays, Illumina)
[12]	*CHRNA5*, *CHRNA3* and *CHRNB4*	15q25,	5,739/5,848; Meta-analysis 7,561/13,818 Caucasian	550 K, 610QUAD4, 317 K + 540S
		5p15, and 6p21		
	HTERT, CLPTM1L			
	HLA region			
[32]	*RAD52*	12p13.33	5,355/4,344 replication 3,359 squamous cell /9,100 Caucasian	Variety of platforms 550, 300, Infinium AB7900 7,700/5,914
[13]	*CHRNA5*, *CHRNA3* and *CHRNB4*	15q25	7,700/5,914 Caucasian	PCR 7,700/5,914
[19]	*CHRNA5*, *CHRNA3* and *CHRNB4*	15q25	11,645/14,954 Caucasian and Asian	Illumina Omni1-Quad and OmniExpress chips
	*HTERT*, *CLPTM1L*	5p15		
[27]	*CHRNA5*, *CHRNA3* and *CHRNB4*	15q25	1,094/1,100 Korean	PCR 1,094/1,100
	*HTERT*, *CLPTM1L*	5p15		
[29]	*TP63*	3q285	Discovery 2,331/3,077 Replication 6,313/6,409 Chinese	--
	*TERT-CLPTM1L*	p15.33		
	*MIPEP*-*TNFRSF19*	13q12.12		
	*MTMR3*-*HORMAD2*-*LIF*	22q12.2		
[30]	*GATA3*	10p14	Discovery 2,331/3,077 Validation 7,436/7,483 Chinese	Affymetrix SNP Array 6.0 TaqMan, iPLEX Sequenom MassARRAY
	*CYP24A1*	20q13.2		
	*PPP2R2B*-*STK32A*-*DPYSL3*	5q32		
	*IL3-CSF2-P4HA2*	5q31.1		
	*AJAP1-NPHP4*	1p36.32		
[31]	*LRFN2*	6p21.17p15.3	Discovery 2,331/4,006 Replication 2,665/11,436	Affymetrix SNP Array 6.0 chips
	*SP4* and *DNAH11*			

Therefore, the challenging question was the degree to which the associations between these chromosome 15q25 variants and lung cancer were due to their effects on smoking intensity, rather than a direct carcinogenic effect. The lung cancer association, though statistically robust, and initially not altered by adjustment for smoking, increasingly appears to be mediated through smoking. However, there is still uncertainty as to the degree with which the association for lung cancer is mediated through genetic risk beyond that attributed to smoking intensity. Saccone *et al*. [13] showed that locus 1 was associated with lung cancer even when controlling for amount smoked per day (odds ratio = 1.31, *P* = 1.99 × 10^−21^), suggesting possible direct genetic effects of locus 1 on this cancer, at least in the presence of smoking. Spitz *et al*. [16] noted that the lung cancer risk associated with the variant genotype was highest in the lightest smokers (<20 cigarettes per day) and younger patients (<61 years), arguing a role for genetic susceptibility in these lesser-exposed groups. Furthermore, they [16] were not able to implicate this locus as a risk factor in other smoking-related cancers (bladder and renal), suggesting genetic effects on both smoking behavior and lung cancer risk.

Wang *et al*. [17] demonstrated that each copy of chromosome 15q risk alleles was associated with increased cigarette consumption of 1.0 cigarette per day at rs12914385 and 0.9 cigarettes per day at rs8042374 with, and concluded that these modest differences in smoking behavior were sufficient to account for the 15q25 association with lung cancer risk. However, it could also be argued that cigarettes per day is not a sufficient proxy for carcinogen exposure [18].

Truong *et al*. [19] used data from 21 case-control studies (nine in North America, eight in Europe and four in Asia) and replicated the association between chromosome 15q25 SNPs and lung cancer risk (Table 1) in white ever smokers (odds ratio = 1.26, CI:1.21-1.32, P-trend = 2 × 10^(-26)^) and also confirmed that this association was higher at younger age of onset (*P*-trend = 0.002), whereas no association was found in never smokers or in Asian participants. Spitz *et al*. [16] found no elevated risk associated with these variants in over 547 lifetime never smoking patients with lung cancer. Subsequent meta-analyses of never smokers with lung cancer (Galvan and Dragani [20] in >1,000 never smokers and >1,800 controls; and Wang *et al*. [17] in 2,405 patients and 7,622 controls) replicated the lack of any statistically significant association with this locus in never smokers.

Other top hits identified in the GWAS have also been replicated. A number of well-designed GWAS and meta-analyses have implicated variants at the 5p15.33 locus in cancer risk at several different sites, including lung cancer in both white and Asian patients [21]. Truong *et al*. [19] confirmed the significant association in white patients for rs2736100 in the chromosome 5p15 locus. Both Troung *et al.*[19] and Landi *et al.*[12] noted a histology-specific role of rs2736100 in adenocarcinoma. This locus was also recently implicated in lung cancer risk in African American patients [22]. There is biologic plausibility for this finding because mean relative telomere length has been associated with four genetic variants of the *hTERT* gene, including rs2736100 [23], and *TERT* gene amplification is responsible for *TERT* mRNA overexpression in a majority of lung adenocarcinomas [24]. Cleft lip and palate transmembrane protein 1-like (*CLPTM1L*) gene also resides in this region of chromosome 5 for which copy number gain has been found to be the most frequent genetic event in early stages of non-small cell lung cancer. James *et al*. [25] demonstrated increased CLPTM1L expression in lung adenocarcinomas and protection from genotoxic stress-induced apoptosis and concluded that anti-apoptotic CLPTM1L function could be another mechanism of susceptibility to lung tumorigenesis. A third region implicated by GWAS in susceptibility to lung cancer in Caucasians is the human leukocyte antigen region at chromosome 6p21 [10,26].

The association with SNPs in the 5p15 and 15q25 regions was confirmed in a Korean population with similar magnitude of effect as reported for other ethnic groups, but there was no association with the 6p locus [27]. Likewise, the effect of the 5p15 SNP was significant only for adenocarcinoma. Truong *et al.*[19] noted no effect for the chromosome 15q locus, but replicated the association with the 5p locus in an Asian population. A Japanese study [28] confirmed the finding at 5p15.33. There have been several GWAS in Chinese populations. Hu *et al*. [29] replicated findings of significance in both 3q28 (*TP53*) and at the 5p13 locus (Table 1). They also reported significance at two additional loci, 12q12 and 22q12 (Table 1). In an attempt to identify additional susceptibility loci in Chinese patients with lung cancer, Dong *et al*. [30] reported genome-wide significance for three additional lung cancer susceptibility loci at 10p14 (close to *GATA3*), 5q32 in *PPP2R2B-STK32A-DPYSL3*, and 20q13.2 in *CYP24A1*. They also found additional associations for rs247008 at 5q31.1 (*IL3-CSF2-P4HA2*), and rs9439519 at 1p36.32 (*AJAP1-NPHP4*). There was suggestive evidence for interactions with smoking dose. Jin *et al*. [31] noted that genetic variants at 6p21.1 and 7p15.3 were associated with risk of multiple cancers in Han Chinese patients, including lung cancer. Finally, Shi *et al*. [32] reported that a locus on *RAD52*, involved in DNA double-strand break repair and homologous recombination, influenced risk of squamous cell lung cancer but not other cell types.

It is likely that many more common variants can be anticipated to contribute to lung cancer risk, although with effect sizes too small to reach significance in genome-wide analyses. It has been argued that there are diminishing returns in predicting disease risk from common marker SNPs, and greater effort should be spent investigating functional relevance of the GWAS findings. For example, evaluating the effect that SNP variation has upon expression and activity of nicotinic receptors can be explored by taking advantage of animal and cellular models of *CHRNA3* and *CHRNA5* knock-out animals [33,34]. Studies of cell lines and primary lung cancers can provide insights into the effects of these variants on proliferation and apoptosis; one such study suggested a role of a proteosome gene in this region beyond the effects of nicotinic receptors [35]. Emerging metabolomic markers may provide useful biomarker dosimeters of smoking damage relative to carcinogenesis. Certainly, multiple strategies are needed to further tease apart these complex relationships [18].

### Overlap in genetic risk factors for lung cancer and chronic obstructive pulmonary disease

Lung cancer and chronic obstructive pulmonary disease (COPD) result from the combined effects of smoking exposure and genetic susceptibility. Tobacco smoke exposure has been responsible for 80% of lung cancers, however only 15% to 20% of chronic smokers develop lung cancer or COPD. Approximately 50% to 90% of smokers with lung cancer also have COPD. Studies have shown that COPD is an independent risk factor for lung cancer among Caucasians and African Americans, conferring a four- to six-fold increased risk. Over the past few years, several lung cancer risk models have been developed [36-40], some of which included pulmonary diseases such as COPD and pneumonia. Consistently, the inclusion of COPD in the models leads to improvement of the discriminatory power and good calibration [41]. The model with the highest discriminatory power reported to date is the extended Prostate, Lung, Colorectal and Ovarian lung cancer risk model [37], which also includes COPD. This dual susceptibility indicates a link between the processes that induce COPD and lung cancer.

Results from recent GWAS suggest a possible overlap in the genetic risk factors predisposing smokers to lung cancer and COPD. Several regions in the genome associated with lung cancer and/or COPD have been identified, including chromosome 1q21, 4q22, 4q24, 4q31, 5p15, 5q32, 6p21, 6q24, 15q25 and19q13 [9,10,41-49]. Several important genes mapping to those regions have also been identified as significant players in the pathogenesis of lung cancer and/or COPD (Table 1), and many of these loci overlap. For example, a variant in the *FAM13A* gene has been reported to have a protective effect in COPD and lung cancer [49]. *CHRNA3/5* (15q25) was reported to be associated with both COPD and lung cancer [10,48,49] through its effects on both smoking exposure and COPD. Using mediation analysis, Wang *et al*. [50] reported that COPD is a mediating phenotype that could partially explain the effect of smoking exposure on lung cancer. These findings suggest the presence of shared susceptibility mechanisms for these two smoking-related diseases. Such susceptibility may also be mediated through receptors expressed on the bronchial epithelium that implicate molecular pathways underlying both COPD and lung cancer [51]. To date, most of the lung cancer and COPD genetic studies have been conducted independent of each other, which has contributed to the mediating effect of one disease over the other being overlooked [52].

### Epigenetic screening and diagnostic markers for lung cancer

Epigenetics is classically defined as the study of changes in downstream phenotypes or gene expression that cannot be attributed to changes in DNA and is heritable. Another refined definition is that epigenetics concerns structural changes in chromosomal regions that are not related to changes in DNA that mark altered activity states [53]. Two major types of epigenetic regulation are DNA methylation and histone modification, both of which are known to modulate gene expression. Given that the abundance of molecular biomarkers in this field have been DNA methylation-based, this section will focus on DNA methylation studies that hold the potential to impact early lung cancer detection (Table 2a).

**Table 2:** Methylation-, gene-expression- and miRNA-based biomarkers for risks and early detection of lung cancer

**Reference**	**Sample type**	**Genetics/genomics platform**	**Clinical settings**	**Key findings**
**a) Epigenetic biomarkers**				
[59]	Sputum, lung tissue, biopsies	MSP	Lung tissue, precursor lesions and bronchial biopsies from patients with SCC and sputum from individuals with suspicion of lung cancer	CDKN21 hypermethylation more often observed in patients with cancer than with no cancer
[66]	Paired serum and lung tissue	MSP	Lung tissue and serum from patients with NSCLC and control	73% of patients had serum DNA that reflected aberrant methylation in their tumors, specifically in CDKN2A, MGMT, DAPK, GSTP1
[60]	Paired sputum and lung tissue	MSP	Lung tissue and sputum from smokers with SCC	CDKN2A and MGMT were hypermethylated in both sputum and tumor of patients at time of diagnosis
[72]	Bronchial epithelial cells, blood lymphocytes, lung tissue	MSP	Paired blood and bronchial epithelial samples from smokers/non-smokers with pre-neoplastic lesions and neoplastic lesions from individuals with NSCL versus controls	ECAD and DAPK more likely to be methylated in smokers’ peripheral lymphocytes or bronchial epithelium and never methylated in non-smokers
[58]	Peripheral blood leukocytes	Illumina Beadchip and Pyrosequencing	Smokers with recently diagnosed SCLC and controls	Forty-three CpG sites were differentially methylated between SCLC and controls, and nine of these, validated by pyrosequencing, could discriminate SCLC with AUC of 0.86
[71]	Paired serum and lung tissue	MSP	Paired serum and lung tissue samples from individuals with lung cancer and controls	Six-gene serum panel that discriminated patients with lung cancer with 75% sensitivity and 73% specificity
**b) Transcriptomics biomarkers**				
[76]	Bronchial brushing, large airway epithelium	Affymetrix array	Bronchial brushings of cytologically normal large airway eptihelium obtained from smokers undergoing bronchoscopy for suspicion of lung cancer	Eighty gene airway biomarker with >80% diagnostic sensitivity and specificity, and 95% sensitivity and negative predictive value when biomarker is combined with cytology collected at bronchoscopy
[78]	Bronchial brushings from normal airway bronchial epithelial cells	(StaRT)-PCR	Normal bronchial epithelial cells of patients with lung cancer and non-lung cancer controls	Fourteen gene airway biomarkers of antioxidant, DNA repair and transcription factor genes with performance in a test AUC >0.84 and an accuracy of 80%
[86]	Peripheral blood mononuclear cells	cDNA array	Blood collection from smokers with newly diagnosed lung cancer confirmed by histopathology	twenty-nine-gene blood signature with >80% sensitivity and specificity
[79]	Bronchial brushing from airway epithelium	Affymetrix array	Bronchial airway brushings of cytologically normal epithelium from smokers with and without lung cancer or premalignancy	Gene-expression signature of PI3K signaling pathway activation was differentially expressed in airways of smokers with lung cancer or dysplasia and was reversible with chemopreventive therapy
[88]	Whole blood	Sentrix whole genome bead chips WG6 (Illumina)	PAX gene-stabilized blood samples from three independent groups consisting of patients with NSCLC and controls	Genes differently expressed in whole blood of patients with NSCLC and controls were used to build a diagnostic classifier with AUC >0.82
[85]	Saliva	Affymetrix array	Whole saliva collected from untreated patients with lung cancer with matched cancer-free controls	Seven highly discriminatory transcriptomic salivary biomarker with AUC = 0.925 with >82% sensitivity and specificity
**c) MicroRNA biomarkers**				
[93]	Sputum	RT-qPCR	Sputum from patients with squamous lung cancer and healthy controls	Three miRNA diagnosed stage I squamous cell lung cancer with AUC = 0.87
[94]	Sputum	RT-qPCR	Sputum from patients with lung adenocarcinoma and healthy controls	Four miRNA diagnosed stage I lung adenocarcinoma with AUC = 0.90
[107]	Serum	Genoexplorer microRNA expression system	Serum from patients with lung cancer versus healthy controls	Two miRNA discriminated individuals with early stages NSCLC with AUC = 0.77
[103]	Serum	Taqman Low Density Arrays RT-qPCR	Serum from asymptomatic patients with NSCLC and healthy smokers. Patients were screened by low-dose CT and sera were collected at the time of diagnosis before the surgery	Thirty-two miRNA predicted risk of developing lung cancer in asymptomatic high-risk individuals with an accuracy of 80%
[109]	Plasma	Taqman Low Density Arrays RT-qPCR	Multiple plasma samples were collected before and at the time of disease, from two independent spiral CT-screening trials	Fifteen miRNA predicted the risk of lung cancer with AUC = 0.85 and 13 miRNA diagnosed lung cancer in undetermined CT nodules with AUC = 0.88
[110]	Plasma	RT-qPCR	Plasma from patients with lung cancer versus healthy controls	Four miRNAs discriminated patients with NSCLC with AUC = 0.93
[105]	Serum	RT-qPCR	Serum from patients with lung cancer versus healthy controls	Ten miRNAs discriminated patients with NSCLC with AUC = 0.97

DNA methylation is an epigenetic mechanism marked by the joining of a methyl group to a cytosine base to form 5-methylcytosine, typically at a CpG dinucleotide near or within a CpG island. When CpG dinucleotides are methylated to a high degree in the promoter region of a gene, that gene’s expression is usually down-regulated as a result. This is one way that cells can regulate which genes are expressed (Figure 2) and is a mechanism utilized during cell and tissue differentiation during development [54]. Aberrant hypermethylation of oncogenes or hypomethylation of tumor suppressor genes (Table 2a) is one way that transcriptional regulation can spiral out of control in cancer cells [55].

Genome-wide methylation profiling has been used to identify altered methylation patterns in lung cancer tissue (including genes such as *CDKN2A*, *RASSF1A*, *ARHI*, *MGMT* and *RARβ*) [56,57], but so far only one larger scale study has shown the possibilities of identifying methylation biomarkers for the diagnostic or screening setting in noninvasive biospecimens utilizing microarray-based technologies. In this study, nine CpGs were able to discriminate between lung cancer cases and controls with an area under the receiver operator characteristic curve (AUC) of 0.86 [58]. The vast majority of current methylation studies that could be useful for screening and diagnostic tests remain at a candidate gene or gene panel level analysis (Table 2a).

Belinsky *et al*. [59] originally identified the hypermethylation of *CDKN2A* in lung tumors but within the same study also examined the sputum of 33 people who smoked. In this small initial study, eight patients had sputum with methylated *CDKN2A* detected by methylation-specific polymerase chain reaction (MSP). Of those, three were diagnosed with lung cancer at the time of sputum collection and one other would develop lung cancer a year later [59]. Work on identifying *CDKN2A*, as well as *MGMT*, as a measure of cancer risk and diagnosis was expanded in a 21-patient study of matched sputum and squamous cell carcinoma (SCC) samples as well as sputum samples from 32 patients evaluated for possible lung cancer. This study was able to significantly improve cancer detection and risk using the methylation status of the two genes compared to cytology alone, with 100% of patients with SCC displaying methylation of one or both of these genes. More importantly, these genes were aberrantly methylated up to three years before diagnosis [60]. By looking at the sputum of lung cancer-surviving smokers, cancer-free smokers and never smokers, then adjusting for age and smoking duration, *MGMT*, *RASSF1A*, *DAPK* and *PAX5α* were also identified as being significantly differently methylated in the lung cancer survivors. This indicates that aberrant methylation of a panel of candidate genes could identify patients with higher risk of lung cancer (lung cancer-surviving smokers had a 6.2-fold higher odds of having three or more of these genes methylated in sputum) [61]. Other genes that have been identified in the sputum with aberrant methylation associated with increased risk for lung cancer include *ASC/TMS1* (increased odds in cancer patients from 7.2 to 28.6) [62], *GATA4*, *GATA5* and *PAX5β* (6.5-fold increase in cancer risk with methylation of three or more genes) [63]. Recently, a larger panel of 31 genes in the sputum was used to identify signatures of stage I lung cancer. It had >70% accuracy and could predict which smokers had cancer between 3 and 18 months before clinical diagnosis (AUC of 0.71 and 0.77 for the two cohorts in the study) [64].

Other potential distal sites for assessing lung cancer risk using methylation markers include the serum, plasma and blood leukocytes. Based on evidence that DNA from tumor cells can be found freely in circulating serum [65], Esteller *et al*. [66] examined the serum, normal lung tissue and tumor tissue from 22 patients with non-small cell lung cancer (NSCLC). They found that 73% of patients had serum DNA that reflected hypermethylation events found in their tumors. Specifically using MSP, they looked at methylation of *CDKN2A*, *MGMT*, *DAPK* and *GSTP1*, genes whose aberrant methylation profiles have already been shown to associate with lung cancer risk or diagnosis [66]. A larger study with a cross-section case-control design looked at the serum from 200 patients, 91 of whom had lung cancer, 100 had non-malignant lung disease, and nine had some other malignant disease. *RARβ*, *CDKN2A*, *DAPK*, *RASSF1A* and *MGMT* were examined, and the analysis showed that a patient having methylation of just one gene had an odds ratio of 5.08, meaning they were approximately five times likely to have lung cancer than patients without any methylated genes. This odds ratio increased in patients with two or more genes being aberrantly methylated [67]. Overall, just looking at this limited candidate gene list, almost 50% of patients with lung cancer displayed at least one case of aberrant methylation in their serum. Other genes with aberrant methylation in serum DNA have been found to associate with lung cancer risk, including *TMEFF2*[68], *RUNX3*[69] and CDH13 [70], suggesting that many genes in the serum could signify lung cancer risk and that a larger profile of aberrant methylation could produce a more accurate biomarker for lung cancer risk. The work by Begum *et al*. [71], who looked at methylation profiles of a slightly larger set of 15 genes and then selected the six most sensitive and specific genes for predicting lung cancer risk (*APC*, *CDH1*, *MGMT*, *DCC*, *RASSF1A* and *AIM1*), clearly shows evidence that a more global methylome approach could lead to a more sensitive (75%) and specific (73%) biomarker of lung cancer risk from serum DNA [71]. Methylation events in plasma, specifically in *CDKN2A*, *MGMT* and *RASSF1A*[61], as well as in peripheral blood leukocytes [58] and lymphocytes [72,73], are promising less invasive sites for assessing lung cancer risk through measuring DNA methylation differences.

### Transcriptomic biomarkers for screening and diagnosing lung cancer

Gene-expression profiling or transcriptomics has been used to delineate disease classification, improve diagnostic accuracy, identify new molecular targets for drugs and provide new biological insights into lung cancer. High-throughput technologies, such as microarray, and sequencing platforms allow the measurement of thousands of genes simultaneously, to look for different pattern changes across subsets that help characterize a particular physiological state or clinical phenotype. In this section, we will review the diagnostic and screening transcriptomic biomarkers that have been developed in the airway and blood of at-risk smokers (Table 2b).

#### Airway-based transcriptomic biomarkers for early detection of lung cancer

A number of transcriptomic biomarkers for the early detection of lung cancer have leveraged the so-called field cancerization or field effect paradigm in which abnormalities in gene expression in the normal bronchial mucosa are shared with those found in the tumor. Two genome-wide gene-expression profiling studies identified transcriptomic alterations related to smoking that were found both in the cancer and in the normal lung tissue [74,75]. The first study analyzed both lung SCC compared to the normal epithelium of the bronchi and adenocarcinoma as compared to the normal alveolar lung tissue [74]. The second study focused on SCC and normal bronchial epithelium [75]. Abnormalities in the normal bronchial tissue that were similar to those identified in the tumor were seen in tumor suppressor genes and oncogenes, as well as different functions such as xenobiotic metabolism and redox stress, matrix degradation, and cell differentiation.

Based on these studies, a number of groups have been using a comparatively easily available specimen, airway epithelial cells through bronchial brushings, to measure the changes in gene expression associated with lung cancer. An 80 gene-expression-based biomarker was developed in mainstem bronchial airway epithelial cells that can serve as a sensitive and a specific biomarker for diagnosing lung cancer among smokers undergoing bronchoscopy for suspected disease [76]. Importantly, combining the gene-expression biomarker with cytology obtained at bronchoscopy resulted in 95% sensitivity and 95% negative predictive value, enabling the physician to avoid unnecessary further invasive procedures in those smokers without lung cancer. Furthermore, the biomarker was shown to be associated with lung cancer diagnosis independent of clinical and radiographic risk factors for disease, although the study was limited in terms of the clinical and radiographic risk factors that were modeled (for example, COPD positron emission tomography scan results not included) [77]. Later, Blomquist *et al*. also reported that a pattern of antioxidant and DNA repair gene expression in normal airway epithelium was associated with lung cancer [78]. They identified a signature of 14 genes that discriminates cases versus controls with an AUC of 0.84 and an accuracy of 80%.

Beyond diagnosing lung cancer, airway gene expression has also been used to identify molecular pathways that are deregulated in the bronchial airway of smokers with or at risk for lung cancer [79]. A gene-expression signature of phosphoinositide-3-kinase signaling pathway was differentially activated in the cytologically normal bronchial airway of both smokers with lung cancer and smokers with pre-malignant airway lesions [76]. Furthermore, that study found that the PI3K pathway gene-expression signature reverses back to baseline in those patients whose dysplastic lesions regress upon treatment with the candidate lung cancer chemoprophylaxis agent myoinositol. As airway epithelial cell dysplasia is a pre-neoplastic event in lung carcinogenesis, these data suggest both that PI3K pathway activation is an early and reversible event during lung carcinogenesis and, more broadly, that bronchial airway epithelial cell gene expression reflects carcinogenic processes that precede the development of frank malignancy [79]. This suggests that alterations in airway gene expression are an early and potentially reversible event in the process of lung carcinogenesis that could potentially be used to guide personalized approaches to lung cancer chemoprevention.

Leveraging the microarray dataset of airway epithelium from smokers with and without lung cancer [76], Wang *et al*. [80] provided additional insight into the molecular pathways altered in the airway of smokers with lung cancer. They identified that the antioxidant response pathway, regulated by the transcription factor nuclear factor erythroid-derived 2-like 2, was down-regulated in the airway of smokers with lung cancer. Furthermore, they identified potential polymorphisms in the promoter regions of the antioxidant genes that may associate with decreased airway gene expression in response to tobacco smoke.

With the emergence of next-generation sequencing as a more robust tool for transcriptomic profiling, Beane *et al*. sequenced the RNA from bronchial airway epithelial cell brushings obtained during bronchoscopy from healthy never smokers, current smokers and smokers with and without lung cancer undergoing lung nodule resection surgery [81]. There was a significant correlation between the RNA-sequencing gene-expression data and Affymetrix microarray data generated from the same samples (*P* <0.001), although the RNA-sequencing data detected additional smoking- and cancer-related transcripts whose expression was not found to be significantly altered when using microarrays.

Over the past several years, a number of studies have attempted to move transcriptomic profiling of the airway in at-risk smokers to biosamples that are less invasive and more easily collected in population-based studies. Two separate groups have demonstrated that the buccal mucosa gene-expression response to smoking mirrors that seen in the bronchial airway (one study using punch biopsies of the cheek [82] and the second using buccal scrapings [83]). Both studies were limited to healthy smokers and did not assess the relationship of bronchial and buccal gene expression within the same individual. More recently, Zhang *et al*. [84] demonstrated a strongly concordant gene-expression response to smoking in matched nasal and bronchial samples from active smokers. These studies raise the exciting possibility that buccal and nasal swabs could be used as a surrogate to bronchial brushings for a relatively noninvasive screening or diagnostic tool for individual susceptibility to smoking-induced lung diseases. Additionally, Zhang *et al*. [85] profiled salivary transcriptomes of recently diagnosed and untreated smoker and non-smoker patients with lung cancer and matched cancer-free controls. The study led to the discovery of seven highly discriminatory transcriptomic salivary biomarkers with 93.75% sensitivity and 82.81% specificity in the pre-validation sample set. Data suggest that lung cancer transcriptomic biomarker signatures are present in human saliva, which could be clinically used to discriminate patients with lung cancer from cancer-free controls.

#### Blood-based transcriptomic biomarkers for early detection of lung cancer

Although the development of a gene-expression biomarker in blood that can be collected in a noninvasive manner is highly attractive, studies have been relatively limited by the degradation of circulating mRNA in serum and plasma. However, gene-expression alterations identified in lung tumors have been identified in circulating white blood cells by a number of groups. Showe *et al*. analyzed gene expression in peripheral blood mononuclear cell samples of current or former smokers with histologically diagnosed NSCLC tumors [86]. They identified a 29-gene signature that separates patients with and without lung cancer with 86% accuracy (91% sensitivity, 80% specificity). Accuracy in an independent validation set was 78% (sensitivity of 76% and specificity of 82%). Rotunno *et al.* analyzed gene expression of lung tissue and peripheral whole blood collected using PAXgene blood RNA tubes from patients with adenocarcinoma and controls to identify dysregulated lung cancer genes that could be tested in blood to improve identification of at-risk patients in the future [87]. Zander *et al*. further investigated the validity of whole-blood-based gene-expression profiling for the detection of patients with lung cancer among smokers from three different datasets. They showed that RNA-stabilized whole-blood samples can indeed be used to develop a gene-expression-based classifier that can be used as a biomarker to discriminate between NSCLC patients and controls [88].

### miRNA biomarkers for the early detection of lung cancer

MicroRNAs are recently discovered small molecules that play an important role in regulating gene expression. These noncoding RNAs, in their final active form, are usually 22 nucleotides in length and target specific parts or mRNA sequences, usually found in the 3′ untranslated regions of mRNA, which either prevent translation or promote mRNA degradation, and lead to down-regulation of specific genes [89]. Because miRNA are relatively more stable than mRNA [90], any miRNA profiles of lung cancer risk or diagnosis are likely to be more accurate when moving from the bench to the clinic. This review will focus on large-scale miRNA studies that have been performed in airway, sputum and blood for early lung cancer detection (Table 2c).

#### In bronchial tissue

By global profiling of miRNA in pre-malignant airway lesions, 69 miRNA were found to evolve in high-risk patients from a pre-invasive stage to a higher stage in the multistep process of lung carcinogenesis. The expression profiles of 30 and 15 miRNAs were able to discriminate low-grade lesions from high-grade ones including or not invasive carcinoma [91]. While this data suggests that airway miRNA expression may serve as an early detection biomarker, this study was limited to bronchial biopsies of pre-malignant airway lesions, which are relatively invasive. As with the gene-expression studies outlined above, more microRNA profiles in airway epithelial brushings are needed to advance the field.

#### In sputum

Given the relative stability of miRNA in biological specimens, a number of groups have explored the utility of miRNA-based biomarkers in sputum samples. Xie *et al*. [92] showed that miRNA profiles in the sputum could be used to identify NSCLC. More recently, two studies were also able to identify and distinguish miRNA profiles that could do early detection of SCC [93] or adenocarcinoma [94]. Both studies included a test set and a validation set. A SCC signature of three miRNAs diagnosed the presence of a stage I SCC in patients’ sputum with a sensitivity of 73%, a specificity of 96% and an AUC of 0.87 in the test set [93]. The adenocarcinoma signature composed of four miRNA detected patients with stage I adenocarcinoma with a specificity of 81%, a sensitivity of 92% and an AUC of 0.90 [94]. There was no overlap between the two signatures in sputum. In total, seven different miRNAs were identified in these two signatures and these miRNAs could be risk factors for lung cancer and be used to diagnose lung cancer.

#### In blood

The relative stability of miRNA has prompted numerous groups to explore the potential utility of a blood-based miRNA biomarker for early detection of lung cancer. Ten of these have been specifically looking for circulating miRNA in plasma or serum, whereas five studies have examined miRNA expression profiles in whole blood [95-99].

Among the whole-blood miRNA studies, one study took a candidate approach by analyzing the expression of let-7a in the blood of patients with NSCLC [97]. The other four studies screened the expression of larger panels of miRNA in a small number of patients (range of 10 to 28 patients per group), not including any validation set [95,96,98,99]. These studies identified an miRNA signature discriminating between patients with lung cancer and healthy controls with a sensitivity and specificity ranging from 86% to 98% and from 88% to 100%, respectively, using cross-validation within training set. Keller *et al*. [96] have applied next-generation miRNA-sequencing to whole blood to identify miRNAs associated with lung cancer. Using ultra-deep (approximately 25 million reads per sample of small RNA) sequencing of blood samples from 10 patients with NSCLC and 10 healthy individuals, they were able to identify seven entirely novel miRNAs (not annotated in miRBase at the time) that were significantly altered in patients with cancer [96]. This relatively small study demonstrates the potential resolution that miRNA-sequencing could provide in discovering entirely new biomarkers for lung cancer.

Seven studies analyzed miRNA expression in serum [100-106] and three in plasma [107-109]. Six out of the ten studies included a validation set and four of the same six studies described the performance of the test, that is, sensitivity, specificity and/or AUC [100,101,103,105,107,108]. Notably, only three studies included samples at earlier time points than diagnosis [101,102,107], which is required for evaluating miRNAs as a risk or screening biomarker. Boeri *et al*. identified miRNA signatures that predict lung cancer development and prognosis [107]. They analyzed miRNA expression in 38 patients with lung cancer from the INT-IEO cohort (training set) and 53 from the MILD trial (validation set). With a signature composed of a ratio of 15 miRNAs, they could predict risk of lung cancer in patients with nodules in the CT screening with a sensitivity of 80%, a specificity of 90% and an AUC of 0.85. A signature composed of a ratio of 13 miRNAs was able to diagnose lung cancer in undermined CT-screened lung nodules with a sensitivity of 75%, a specificity of 100% and an AUC of 0.88. The study of Boeri *et al*. [107] is the only work so far directly addressing the role of biomarkers for the work-up of CT-screened nodules. In addition to requiring further prospective validation, this study might be too complex to apply in practice. Another more recent study by Bianchi *et al*. [101] identified a 34-miRNA profile that could predict which asymptomatic high-risk individuals were likely to develop a lung cancer with an accuracy of 80%. Among the 5,203 high-risk individuals studied, 93 went on to be diagnosed with NSCLC in the first two years of screening. Serum was collected before surgery from 59 of these 93 patients, and serum was also collected from 69 matched control patients who were enrolled in the same study. Using a training set and test set, they were able to identify a 34-miRNA biomarker, one which can better identify lung cancer risk and be more properly used as a screening test [101].

### Free circulating DNA biomarkers

Circulating cell-free DNA (cf-DNA) is a known marker of cancer cell death and an increase in the prevalence of cf-DNA in the blood has been used as a marker to distinguish patients with cancer patients from patients with no cancer [110,111]. In this section, we will review recent efforts to utilize cf-DNA as a diagnostic and screening biomarker for differentiating patients with lung cancer from those without, especially some studies that have been able to identify changes in cf-DNA that can distinguish patients with early stage lung cancer from patients with no cancer.

Utilizing polymerase chain reaction (PCR), Sozzi *et al*. [112] examined the levels of plasma DNA in 84 patients with NSCLC and 43 healthy blood donor controls, and showed over a range of cutoff points that healthy controls could be distinguished from patients with lung tumors with an AUC of 0.844. They showed that, even in patients with stage 1a cancer, the amount of circulating plasma DNA was significantly higher than in the control patients [112]. Although this study showed the use of quantifying cf-DNA to distinguish patients with cancer from healthy controls, only 8 of the 43 controls were smokers, so smoking may have been a confounding effect in the cancer diagnosis. Sozzi *et al*. [113] addressed this by matching 93 control individuals who smoked with 100 patients with NSCLC, and were able to discriminate the patients from controls by concentration of cf-DNA with an AUC of 0.94. They also quantified the risk such that a unit increase in plasma DNA led to a 21% increase in NSCLC risk [113]. Other studies have shown that cf-DNA can distinguish patients with benign lung disease from lung cancer with an AUC of 0.73 [114]. However, in a screening cohort of >1,000 higher risk smoking volunteers, Sozzi *et al*. [115] found that quantification of cf-DNA could not be used to distinguish the individuals who would develop lung cancer from those who did not [115]. Other studies have also confirmed this finding [116].

In terms of the emerging clinical needs (Figure 1), these data argue that cf-DNA may not be an effective marker for screening high-risk smokers, but based on these and many other cf-DNA studies [117-120], it could still play a role in diagnosing whether nodules identified by low-dose CT are either benign or malignant. The field is also progressing towards the identification of screening- or diagnostic-specific markers within lung tumor circulating cf-DNA including methylation markers [68] and genetic mutations such as epidermal growth factor receptor mutations [121-123]. Although these developments are relatively new, the Dawson *et al*. study [124], in which genetic alterations could be identified in cf-DNA that corresponded dynamically with metastatic breast cancer, shows the potential for identifying novel genomic and genetic biomarkers within cf-DNA to better stratify patients [124].

## Conclusions

As CT screening programs for lung cancer proliferate in the post-National Lung Screening Trial era, there is an urgent and growing need to develop and validate biomarkers that can both help identify those smokers at highest risk who are most likely to benefit from screening and help distinguish benign from malignant lesions found on chest imaging. The recent advances in genetics and genomics have ushered in an era of genome-wide studies aimed at identifying molecular biomarkers for diagnosis and risk for lung cancer. While a number of promising genetic, transcriptomic and epigenomic markers have been identified as detailed above, we have yet to see translation from biomarker discovery to clinical application.

A review of these studies reveals several important limitations that will need to be addressed in the coming years if the field is to advance and have a clinical impact. First, molecular biomarkers discussed in this review will need to be validated in multicenter trials on independent cohorts to demonstrate the validity and generalizability of the biomarker. Importantly, the biomarkers will need to be validated in the clinical setting in which they will be applied. This latter caveat is best addressed at the biomarker development stage, where molecular markers are identified among clinical specimens that reflect the ultimate clinical application (for example, for diagnostic markers, using specimens collected prior to lung cancer diagnosis among patient and controls who present with suspicion of disease). To have clinical utility, these molecular markers will need to demonstrate performance metrics that would alter clinical decision making (for example, having a very high negative predictive value in the diagnostic setting). They will further need to demonstrate that they provide information about cancer risk and/or diagnosis that is independent of clinical and radiographic risk factors that have been well established for disease. The ultimate translation to the clinic, however, will require transitioning to analytical platforms that can be readily applied in the clinic to facilitate physician adoption as part of their standard of care.

## Abbreviations

AUC: Area under the receiver operator characteristic curve; COPD: Chronic obstructive pulmonary disease; CT: Computed tomography; GWAS: Genome-wide association study; MSP: Methylation-specific PCR; RT-qPCR: Reverse transcription quantitative polymerase chain reaction; SCC: Squamous cell carcinoma; NSCLC: Non-small cell lung cancer; SCLC: Small cell lung cancer; SNP: Single nucleotide polymorphism.

## Competing interests

AS is a founder and consultant to AllegroDx Inc. All other authors declare that they have no competing interests.

## Authors’ contributions

All authors contributed to the writing of this manuscript. JB, CM, KH and AS wrote the transcriptomics, epigenetics and miRNA sections. RE and MS wrote the genetics section. JB and KH were responsible for the final design of the figures and tables. All authors read and approved the final manuscript.

## Pre-publication history

The pre-publication history for this paper can be accessed here:

http://www.biomedcentral.com/1741-7015/11/168/prepub
